# The Potential of Eukaryotic Cell-Free Systems as a Rapid Response to Novel Zoonotic Pathogens: Analysis of SARS-CoV-2 Viral Proteins

**DOI:** 10.3389/fbioe.2022.896751

**Published:** 2022-04-19

**Authors:** Franziska Ramm, Srujan K. Dondapati, Hoai Anh Trinh, Dana Wenzel, Ruben M. Walter, Anne Zemella, Stefan Kubick

**Affiliations:** ^1^ Fraunhofer Institute for Cell Therapy and Immunology (IZI), Branch Bioanalytics and Bioprocesses (IZI-BB), Potsdam, Germany; ^2^ Institute of Chemistry and Biochemistry, Freie Universität Berlin, Berlin, Germany; ^3^ Department of Applied Biochemistry, Institute of Biotechnology, Technical University Berlin, Berlin, Germany; ^4^ Faculty of Health Sciences, Joint Faculty of the Brandenburg University of Technology Cottbus–Senftenberg, The Brandenburg Medical School Theodor Fontane, The University of Potsdam, Potsdam, Germany

**Keywords:** eukaryotic cell-free systems, SARS-CoV-2, viral proteins, rapid response, protein analytics, planar lipid bilayer measurements

## Abstract

The ongoing pandemic caused by the novel coronavirus (SARS-CoV-2) has led to more than 445 million infections and the underlying disease, COVID-19, resulted in more than 6 million deaths worldwide. The scientific world is already predicting future zoonotic diseases. Hence, rapid response systems are needed to tackle future epidemics and pandemics. Here, we present the use of eukaryotic cell-free systems for the rapid response to novel zoonotic diseases represented by SARS-CoV-2. Non-structural, structural and accessory proteins encoded by SARS-CoV-2 were synthesized by cell-free protein synthesis in a fast and efficient manner. The inhibitory effect of the non-structural protein 1 on protein synthesis could be shown *in vitro*. Structural proteins were quantitatively detected by commercial antibodies, therefore facilitating cell-free systems for the validation of available antibodies. The cytotoxic envelope protein was characterized in electrophysiological planar lipid bilayer measurements. Hence, our study demonstrates the potential of eukaryotic cell-free systems as a rapid response mechanism for the synthesis, functional characterization and antibody validation against a viral pathogen.

## Introduction

**FIGURE 1:**
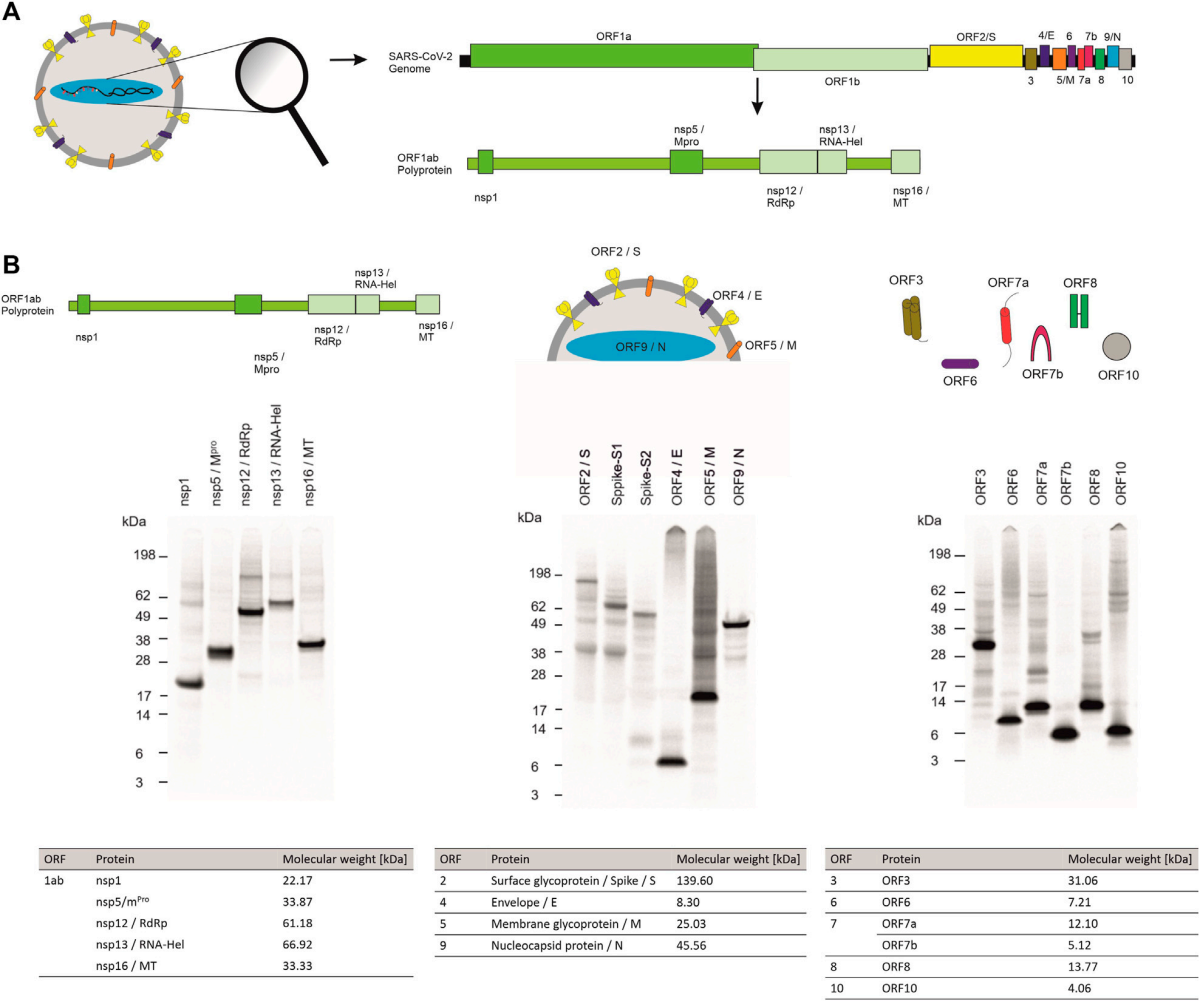
Cell-free synthesis of SARS-CoV-2 proteins in a CHO cell-free system. **(A)** Schematic figure showing the SARS-CoV-2 virus particle and its structure as well as the coding sequences of the genome. **(B)** Qualitative analysis of cell-free synthesized SARS-CoV-2 proteins by autoradiography. Autoradiograph showing ^14^C-leucine labeled proteins synthesized in a CHO system from the translation mixture (TM).

Infectious diseases that are transferred from an animal to a human being, so-called zoonoses, can lead to devastating health issues around the world as can be seen from the example of the severe acute respiratory syndrome coronavirus type 2 (SARS-CoV-2). The close interaction with animals, such as in agriculture and with domesticated animals (pets), the increasing consumption of different meats as well as the intrusion of humans into the natural habitat of animals, causes a high risk for the development of novel zoonoses that might lead to short lived disease outbreaks, epidemics or even pandemics. Standard techniques such as the detection of viral antigens by polymerase chain reactions (PCR) as well as rapid antigen tests accelerate prompt responses such as quarantines and shutting down social contacts. Unfortunately, some pathogens, such as airborne viruses, are persistent and have to be counteracted with vaccines and therapeutics. Consequently, the thorough characterization of the virus itself and its mode of action, including the viral assembly, cell attack, pathogenesis of the underlying disease and characterization of the viral proteins are necessary to tackle these tasks and to identify efficient drugs. It is essential to understand the different modes of action of the individual proteins as each particular component plays a specific role in the viral assembly, host infection and immune invasion as well as viral replication. The novel coronavirus expresses 10 different open reading frames (ORFs) encoding the ORF1ab polyprotein, eight single ORFs corresponding to eight single proteins as well as ORF7 which can be further separated into ORF7a and b which encode two different proteins (Figure 1A) (Kim et al., 2020; Wang et al., 2020; Yoshimoto, 2020). The ORF1ab polyprotein encodes 16 non-structural proteins (nsp) including proteases (nsp3 and nsp5), a RNA-helicase (nsp13) and a RNA-dependent RNA-polymerase (RdRp, nsp12) that are mainly responsible for viral replication (Gao et al., 2020; Jang et al., 2020; Shin et al., 2020; Shu et al., 2020; Yoshimoto, 2020; Zhang et al., 2020). This polyprotein is encoded by the two ORFs ORF1a and ORF1b. A frameshift before the stop codon in ORF1a facilitates the translation to be continued to ORF1b and therefore resulting in the polyprotein ORF1ab (Kim et al., 2020). The other ORFs can be divided into two major classes, namely the structural proteins and the accessory proteins. Structural proteins are well known and well characterized as these proteins assemble to the viral capsid. The core structure of SARS-CoV-2 virus is maintained by the ORF2 surface glycoprotein (S) otherwise known as the Spike protein, the ORF4 envelope protein (E), the ORF5 membrane glycoprotein (M) and the ORF9 nucleocapsid protein (N) (Figure 1A). The accessory proteins of SARS-CoV-2 include membrane proteins like the putative ion channel encoded by ORF3 (Kern et al., 2021), the type I transmembrane protein coded by ORF7a (Rosenthal et al., 2020) as well as the integral membrane protein ORF7b (Schaecher et al., 2008). Accessory proteins ORF6 and ORF8 are known to be involved in the host cell immune invasion and in interferon signaling (Miorin et al., 2020; Flower et al., 2021; Zhang et al., 2021). In some infections the protein encoded by ORF10 is present whilst in others this protein cannot be found which might be caused due to a read through of this ORF (Pancer et al., 2020; Hassan et al., 2021). The proteins encoded by SARS-CoV-2 and their characteristics are summarized in Table 1.

**TABLE 1:** SARS-CoV-2 viral proteins and their characteristics.

ORF	Protein	Short Form	Function
1ab	Nsp1	—	Inhibition of protein translation
	Nsp5	m^Pro^	Protease
	Nsp12	RdRp	RNA-dependent RNA polymerase
	Nsp13	RNA-Hel	RNA-helicase
	Nsp16	MT	Methyltransferase
2	Surface glycoprotein	Spike/S	Binding of host cells
3	ORF3	—	Ion channel
4	Envelope	E	Facilitates assembly
5	Membrane protein	M	Interacts with E, S and N to form stable assembly
6	ORF6	—	Immune invasion
7	ORF7a	—	Virus-host interaction, the type I transmembrane protein
	ORF7b	—	Virus-host interaction, integral membrane protein
8	ORF8	—	Immune invasion
9	Nucleocapsid protein	N	Genome packaging
10	ORF10	—	Unclear

In order to characterize such a versatile set of proteins, a system that can produce and characterize all kinds of proteins is needed. Therefore, we present eukaryotic cell-free systems as a promising methodology for the fast and efficient synthesis and characterization of viral proteins based on the example of SARS-CoV-2 proteins. In cell-free protein synthesis a crude cell lysate rather than viable, intact cells are used, allowing the synthesis of “difficult-to-express proteins” such as membrane proteins or even cytotoxic proteins (Chalmeau et al., 2011; Orth et al., 2011; Henrich et al., 2015; Thoring et al., 2017). With the utilization of a cell lysate, the production of genetically modified organisms becomes obsolete. As a result no high laboratory safety standards are necessary for the cell-free production of toxic and viral proteins. In order to avoid cloning procedures for the generation of templates encoding viral proteins, PCR templates can be used for cell-free synthesis (Sawasaki et al., 2002). Thus, a fast screening of different mutants is possible. Another advantage of some eukaryotic cell-free systems are endogenous microsomal vesicles derived from the endoplasmic reticulum (ER) that are present in the lysate. These vesicles enable post-translational modifications (PTMs) and are a natural surrounding for membrane proteins (Brödel et al., 2014). Here, we demonstrate that the whole set of viral proteins derived from SARS-CoV-2 including membrane proteins, enzymes as well as modulatory proteins can be synthesized in a lysate based on Chinese hamster ovary (CHO) cells (Brödel et al., 2014; Thoring et al., 2017). The synthesis of functional protein was verified by cell-free synthesized nsp1 downregulating the *in vitro* synthesis of a model protein and the demonstration of cytotoxic events as well as single channel events in planar lipid bilayer measurements induced by the cell-free synthesized envelope protein. Further, the cell-free synthesized nucleocapsid protein was used to demonstrate the possibility to quantitatively evaluate the binding of commercially available antibodies. Taken together eukaryotic cell-free systems, including but not limited to the use of CHO lysate, can be applied to characterize viral proteins and might facilitate the screening of antibodies as well as pharmaceuticals and blockers against these viral proteins.

## Results

Viral pathogens such as SARS-CoV-2 induce cytotoxic effects often associated with severe damage to the host cell. This might be one of the major factors in the pathology and disease caused by viruses. A valid system to characterize novel viral pathogens should be able to synthesize and characterize structural as well as non-structural proteins. Therefore, we used a eukaryotic cell-free system to synthesize non-structural, structural and accessory proteins encoded by SARS-CoV-2 (Figure 1A). Qualitative analysis of viral proteins synthesized in a CHO cell-free system showed that all viral proteins tested, could be synthesized. Additionally, multimerization of proteins such as ORF3 and ORF7a as well as ORF8 and ORF10 was visualized and defined cleavage products as seen for Spike proteins and nucleocapsid protein were detected by autoradiography (Figure 1B).

**FIGURE 2:**
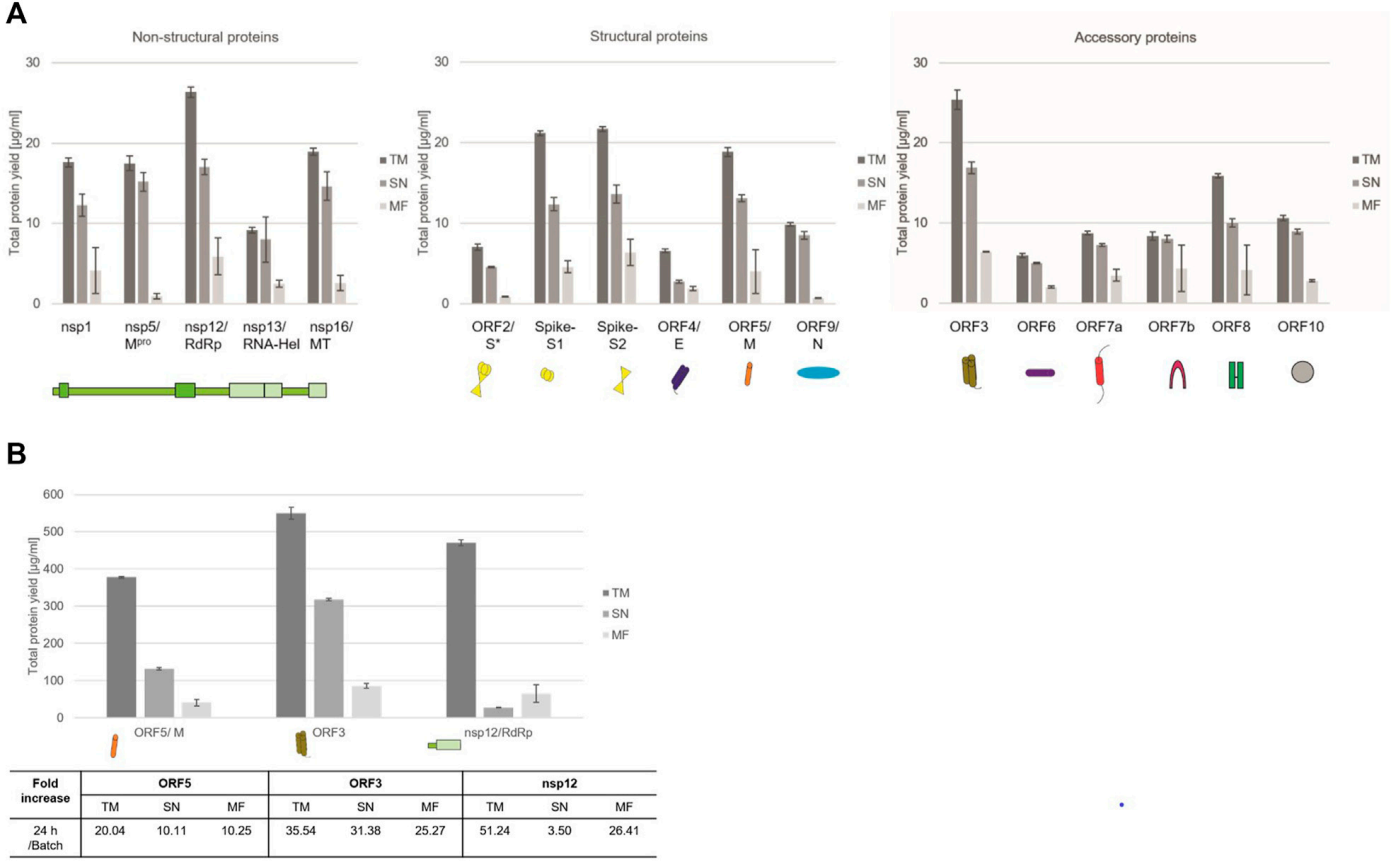
Quantitative analysis of cell-free synthesized SARS-CoV-2 proteins. Viral proteins were synthesized in CHO lysate in **(A)** batch-based reaction and **(B)** a CECF reaction. The fold increase of total protein yield from a batch reaction to a CECF reaction is shown. Quantitative analysis of ^14^C-labeled cell-free synthesized proteins was performed by liquid scintillation counting. Standard deviations were calculated from triplicate analysis. The translation mixture (TM) was separated into the soluble proteins in the supernatant (SN) and the microsomal fraction (MF). The template for the full length Spike protein was based on a PCR-template (*).

Quantitative analysis by hot TCA precipitation and subsequent liquid scintillation verified the acquired data for the qualitative analysis. Total protein yields for non-structural proteins showed that these proteins were mainly present in a soluble form as higher protein yields were detected in the supernatant fraction (SN) compared to the microsomal fraction (MF). Cell-free protein synthesis of the full length Spike protein (ORF2/S) was conducted using a PCR template which resulted in a lower template concentration used. This reduced template concentration led to lower total protein yields. Nonetheless, the high molecular weight protein could be synthesized in an equal amount to the comparably small envelope protein (ORF4/E). The initial data for the synthesis of the accessory proteins showed that all of these proteins could be synthesized as well. The immunomodulatory protein encoded by ORF6 showed the overall lowest protein yield of 6 μg/ml while the transmembrane protein encoded by ORF3 showed the highest protein yields with 25 μg/ml (Figure 2A).

In order to increase the protein yields in a continuous-exchange cell-free (CECF) system, one representative protein of the three protein groups was synthesized for 24 h. The nsp12 coding for an RNA-dependent RNA polymerase (RdRp), the ORF5 membrane glycoprotein and the channel-like ORF3 were chosen (Figure 2B). The protein yields from a batch-based synthesis could be increased by about 20, 35 and 50 fold for ORF5, ORF3 and nsp12, respectively, in a 24 h CECF reaction. Apparently, the nsp12 enzyme was not suitable for a CECF reaction as the soluble protein aggregated in the MF which suggests that a batch-based reaction was more suitable for this enzyme. These data indicate that CFPS offers a platform for the rapid synthesis and analysis of SARS-CoV-2 proteins. As each protein showed different requirements for the cell-free synthesis, the open cell-free system offers an easy way to adapt the synthesis conditions to the need of each individual protein.

**FIGURE 3:**
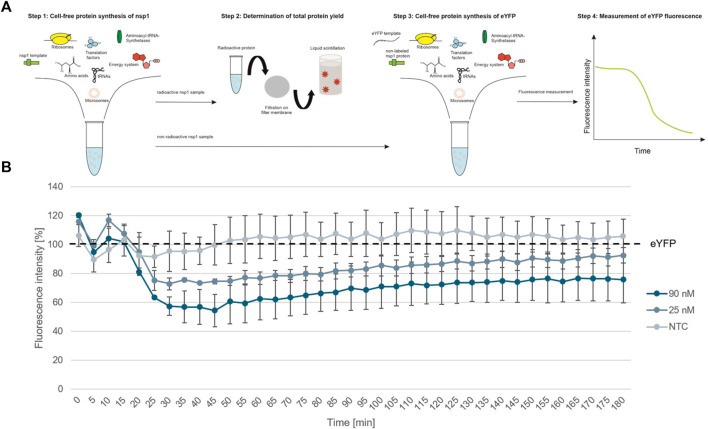
Functional analysis of nsp1. **(A)** Schematic scheme of nsp1 functionality assay. **(B)** Application of cell-free synthesized nsp1 at 90 and 25 nM to the cell-free protein synthesis of the model protein eYFP. Data were normalized to eYFP fluorescence signal. Standard deviation was calculated from two experiments.

To further show the applicability of CFPS as a rapid response system for viral pathogens, we analyzed the individual protein groups and analyzed the functionality of proteins of interest. In a first step, cell-free synthesized nsp1 protein was characterized. This protein is also known as the leader protein responsible for the inhibition of host protein translation (Banerjee et al., 2020; Thoms et al., 2020; Lapointe et al., 2021). It was further shown that nsp1 did not decrease the translation of viral mRNA (Banerjee et al., 2020), thus nsp1 was synthesized without any alterations in the cell-free synthesis scheme. The nsp1 protein was pre-synthesized in a cell-free manner and was added to the cell-free synthesis of the model protein enhanced yellow fluorescent protein (eYFP). Subsequently, the fluorescence intensity of eYFP was measured during a 3 h synthesis time (schematic representation in Figure 3A). The fluorescence signal of eYFP without the supplementation of nsp1 was set as a baseline value of 100%. All other data were normalized to this intensity. In an initial experiment pre-synthesized nsp1 from a CECF reaction, a volume equivalent NTC and ORF6 as a protein control were added to the eYFP synthesis. These data showed that the addition of nsp1 at concentrations of 1000 and 600 nM reduced the fluorescence intensity of eYFP in a concentration dependent manner. 1000 nM of nsp1 led to a complete inhibition of eYFP fluorescence. Unfortunately, the addition of the NTC and ORF6 at 600 nM showed interactions with the eYFP fluorescence. Nonetheless, 600 nM nsp1 induced the highest fluorescence decline (Supplementary Figure S1). Therefore, the reaction conditions were optimized and a batch-based reaction was used. Two concentrations (25 and 90 nM) of nsp1 protein were added to the eYFP synthesis. The NTC was administered in a volume equivalent to the highest nsp1 concentration. Starting after about 20 min reaction time, the fluorescence intensity of the eYFP slowly decreased when nsp1 was supplemented to the reaction but no specific effect could be seen for an NTC supplementation (Figure 3B). A concentration of 90 nM of nsp1 decreased the eYFP fluorescence to less than 60%, while 25 nM nsp1 decreased the eYFP fluorescence to about 75%. This suggested a concentration dependent effect. Strikingly, the inhibitory effect of nsp1 weakened over time so that the fluorescence intensity of eYFP increased again (Figure 3B). Nonetheless, the translation inhibition effect of nsp1 could be shown.

**FIGURE 4:**
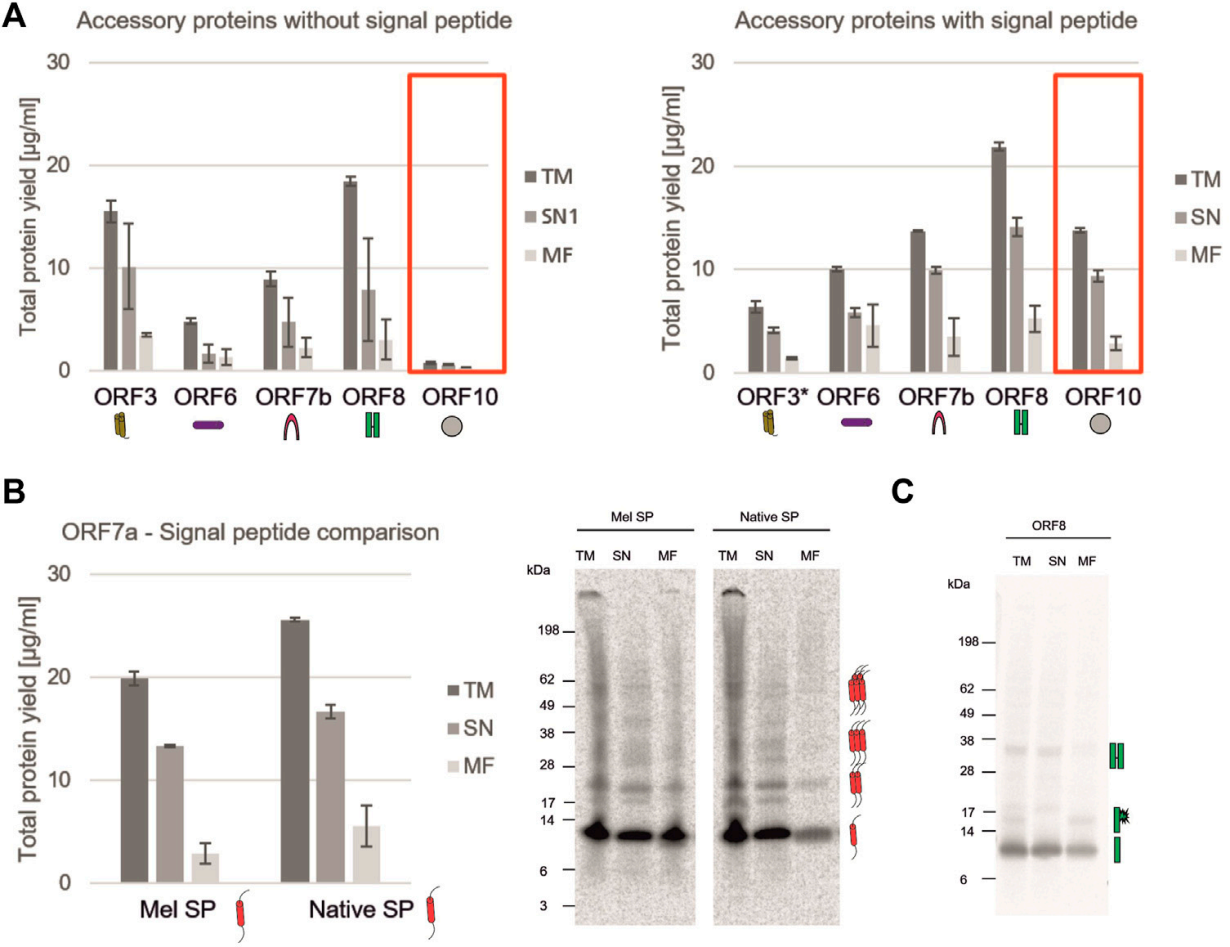
Analysis of SARS-CoV-2 accessory proteins. **(A)** Accessory proteins ORF3, ORF6, ORF7b, ORF8 and ORF10 with and without a Mel signal peptide were synthesized in a CHO batch-based reaction. Quantitative analysis of cell-free synthesized proteins was performed by liquid scintillation counting. Standard deviations were calculated from triplicate analysis. The template for ORF3 with a Mel signal peptide was based on a PCR-template (*). **(B)** ORF7a was synthesized in a CHO batch-based cell-free system with Mel and a native signal peptide. Quantitative analysis of cell-free synthesized proteins as performed by liquid scintillation counting. Standard deviations were calculated from triplicate analysis. Qualitative analysis by autoradiography. **(C)** Autoradiograph showing ORF8 synthesized in a CHO cell-free system. In all experiments, the translation mixture (TM) was separated into the soluble proteins in the supernatant (SN) and the microsomal fraction (MF).

The characterization of non-structural viral proteins is essential for a rapid response to a virus. Nonetheless, the virus needs accessory proteins to stably infect the host. In SARS-CoV-2 these are known to trigger a variety of different interactions within the host such as the involvement in the interferon signaling pathway (Miorin et al., 2020; Yuen et al., 2020), causing an immune invasion (Flower et al., 2021) or acting as membranous channel-like proteins in order to disrupt the host`s cell homeostasis (Yoshimoto, 2020; Kern et al., 2021). As shown in Figure 1, all accessory proteins could be synthesized in a cell-free manner and showed that cell-free protein synthesis can be a tool to rapidly analyze the synthesis of such proteins, to identify optimal synthesis conditions as well as to assess their solubility. In the beginning of a pandemic, the role of such proteins is not yet fully known, therefore we tested whether a signal peptide interferes with a defined protein or might even inhibit the translation of such a protein. Accordingly, ORF3, ORF6, ORF7b, ORF8, and ORF10 were synthesized with and without a Melittin (Mel) signal peptide, which typically allows for the co-translational translocation. As some proteins might inhibit the protein translation machinery, the Mel signal peptide allows for the translocation of the protein into the vesicles present in the cell-free lysates, enabling protein translation. All constructs with and without a signal peptide were fractionated into the soluble proteins (SN) and the proteins in the microsomal fraction (MF). In general, our data indicated that all ORFs were more stably expressed in the presence of a signal peptide (Figure 4A). The NCM-ORF3 construct was based on a PCR template which might have resulted in a lower overall translation efficiency due to a lower template concentration. Strikingly, ORF10 protein translation could not be quantitatively detected without a signal peptide (Figure 4A, red box). In the presence of a Mel signal peptide, protein yields of 13.7 μg/ml were detected for ORF10 suggesting a better translation initiation. ORF7a is known to harbor a native signal peptide as it encodes a type I transmembrane protein (Rosenthal et al., 2020). Comparing the native signal peptide (Nat-SP) with the Mel signal peptide, the native signal peptide showed a higher total amount of protein and a higher protein yield in the MF suggesting efficient targeted embedding into the microsomal membrane. Qualitative analysis showed potential multimerization with the Mel and the native signal peptide. Protein bands of the potential monomer (∼12 kDa), dimer (∼24 kDa), trimer (∼36 kDa) and pentamer (∼60 kDa) could be detected (Figure 4B). The ORF8 protein showed high protein yields with and without a Mel signal peptide. Recent studies have shown that ORF8 contains a sequence homology to the SARS CoV ORF8ab native signal peptide (Tan et al., 2020), indicating that in the Mel-ORF8 construct two signal peptides were present. Hence, later work only focused on the ORF8 template without the Mel signal peptide as it contained the putative native signal peptide. Our data depict that potential SDS-stable multimerization was possible even in the presence of DTT. The autoradiograph also depicts a protein band with a higher molecular weight (∼15 kDa) in the TM and MF fraction but not in the SN fraction indicating a glycosylated ORF8 (Figure 4C). Taken together these data clearly qualify cell-free protein synthesis for the in depth analysis of the translation of viral proteins and the analysis of signal peptide use, protein translocation and translation initiation.

**FIGURE 5:**
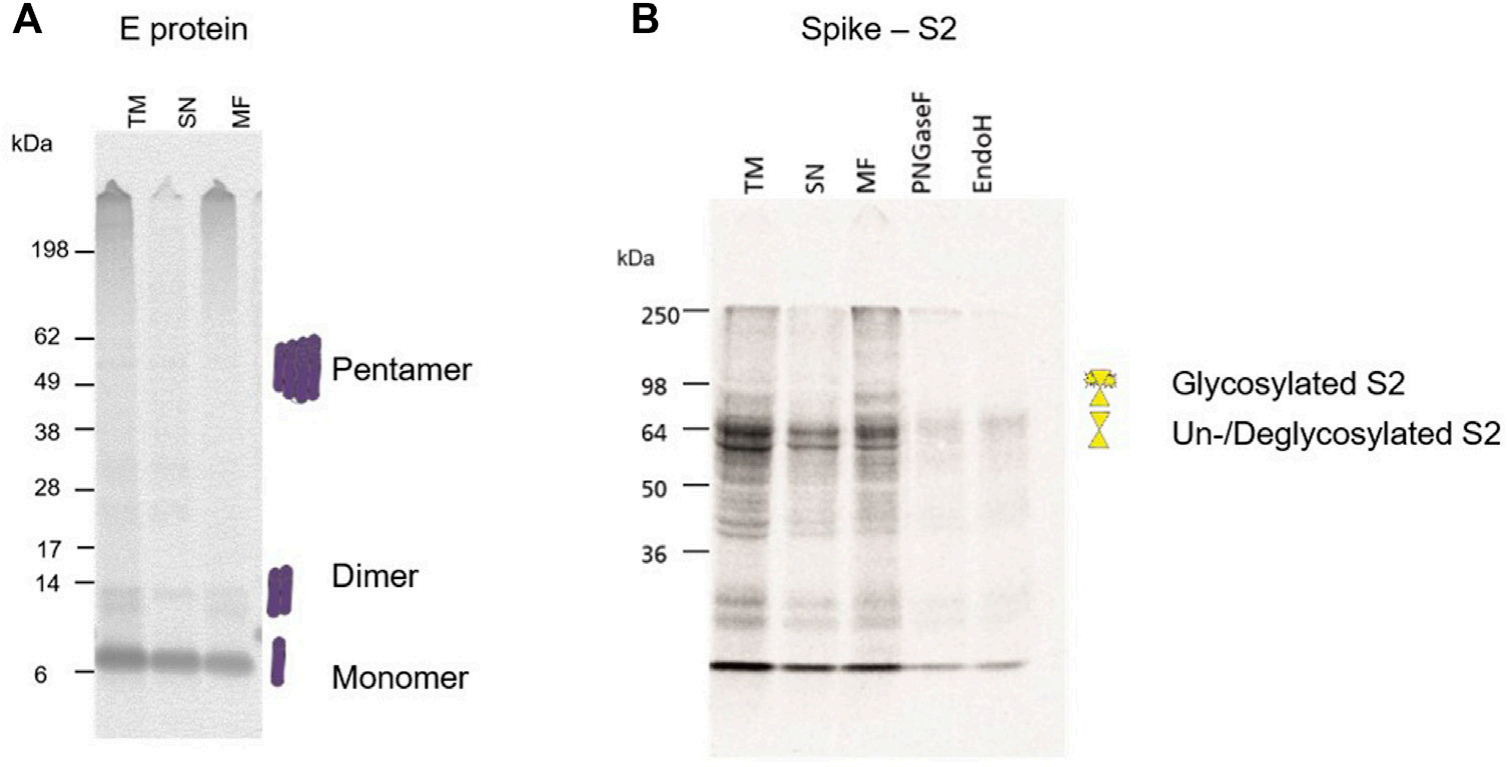
Structural protein analysis of E and S2. **(A)** Autoradiograph showing the envelope protein synthesized in a CHO cell-free system showing multimerization. The translation mixture (TM) was separated into the soluble proteins in the supernatant (SN) and the microsomal fraction (MF). **(B)** Autoradiograph showing the S2 domain from the Spike protein synthesized in a CHO cell-free system. The translation mixture (TM) was separated into the soluble proteins in the supernatant (SN) and the microsomal fraction (MF). The MF was digested with PNGaseF and EndoH.

During a pandemic, structural proteins play a major role in the rapid response to the novel pathogen. Typically, they are used for the detection of the virus itself by PCR techniques as well as antigen detection. Hence, our study aimed to identify the possibility of cell-free synthesized viral antigens for the characterization and detection of structural proteins. As the small envelope protein is reported to form a homo-pentamer (Mandala et al., 2020), the multimerization of the envelope protein was investigated. The multimerization during a batch-based cell-free protein synthesis showed that a monomeric state was preferred but multimers could also be detected (Figure 5A). In comparison to the envelope protein, the surface glycoprotein Spike is a large protein. It is cleaved into the S1 and S2 domain which were individually analyzed. As seen in Figure 1B, the full-length protein as well as the cleavage products can be synthesized in a cell-free manner. On an exemplary basis, the S2 domain was investigated for its N-linked glycosylation. Hence, the S2 domain was synthesized, fractionated and the MF was digested by PNGaseF and EndoH. Autoradiography showed that the TM and MF depicted an additional protein band with a higher molecular weight in comparison to the SN. When the MF was digested with PNGaseF and EndoH, this additional protein band was not detectable anymore, indicating glycosylation in the cell-free system (Figure 5B).

**FIGURE 6:**
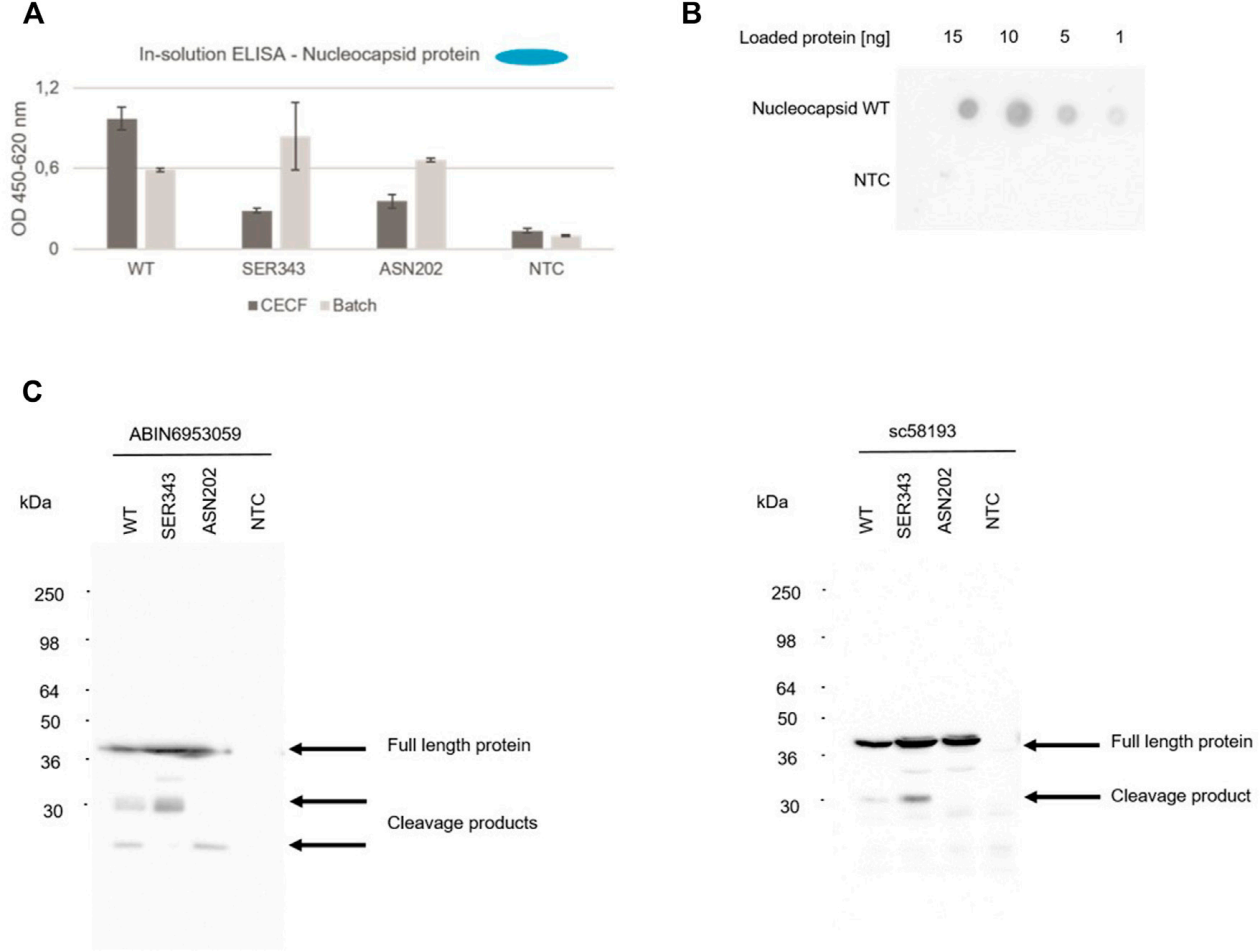
Detection of N protein. The nucleocapsid WT protein as well as both mutants (SER343 and ASN202) were synthesized in a CHO cell-free system. Detection of cell-free synthesized nucleocapsid protein in an in-solution ELISA **(A)**, a Dot Blot **(B)** and Western Blots **(C)**.

The nucleocapsid protein (N) is a major contributor to the structure and humoral response of the virus (Smits et al., 2021) but is also a key structure for diagnostic purposes such as rapid antigen tests (Grant et al., 2021). In this context, an in-solution ELISA was used to detect the cell-free synthesized nucleocapsid wild type (WT) protein as well as two early onset mutants (SER343, ASN202), which were detected in April 2020, in order to validate cell-free synthesis for testing antibodies as well as providing viral antigens for the diagnostic use. N protein, WT as well as mutants, synthesized in a batch and in a 24 h CECF reaction could be detected in an in-solution ELISA (Figure 6A). To further demonstrate the use of cell-free synthesized proteins for the validation of antibodies we performed a quantitative dot blot analysis. N WT protein was blotted onto a nitrocellulose membrane at four different concentrations (15, 10, 5, 1 ng). Our data showed that even concentrations of 1 ng could be detected (Figure 6B). In the early onset of a pandemic antibodies and antivirals against similar viral strains are tested for their efficacy. Here, the detection with an anti-SARS-CoV-2 N antibody (ABIN6953059) as used in the prior experiments was compared to an anti-SARS N antibody (sc58193). Corresponding to the prior data, a Western Blot analysis showed that the N protein was detected by both antibodies. Further, cleavage products could be detected. Strikingly, a protein band at about 30 kDa in the WT and SER343 mutant but not in the ASN202 mutant was identified. The anti-SARS-CoV-2 N antibody showed less by products at an exposure time of 20 s (Figure 6C). These data highlight the efficiency of cell-free protein synthesis for the validation of antibodies detecting viral proteins.

**FIGURE 7:**
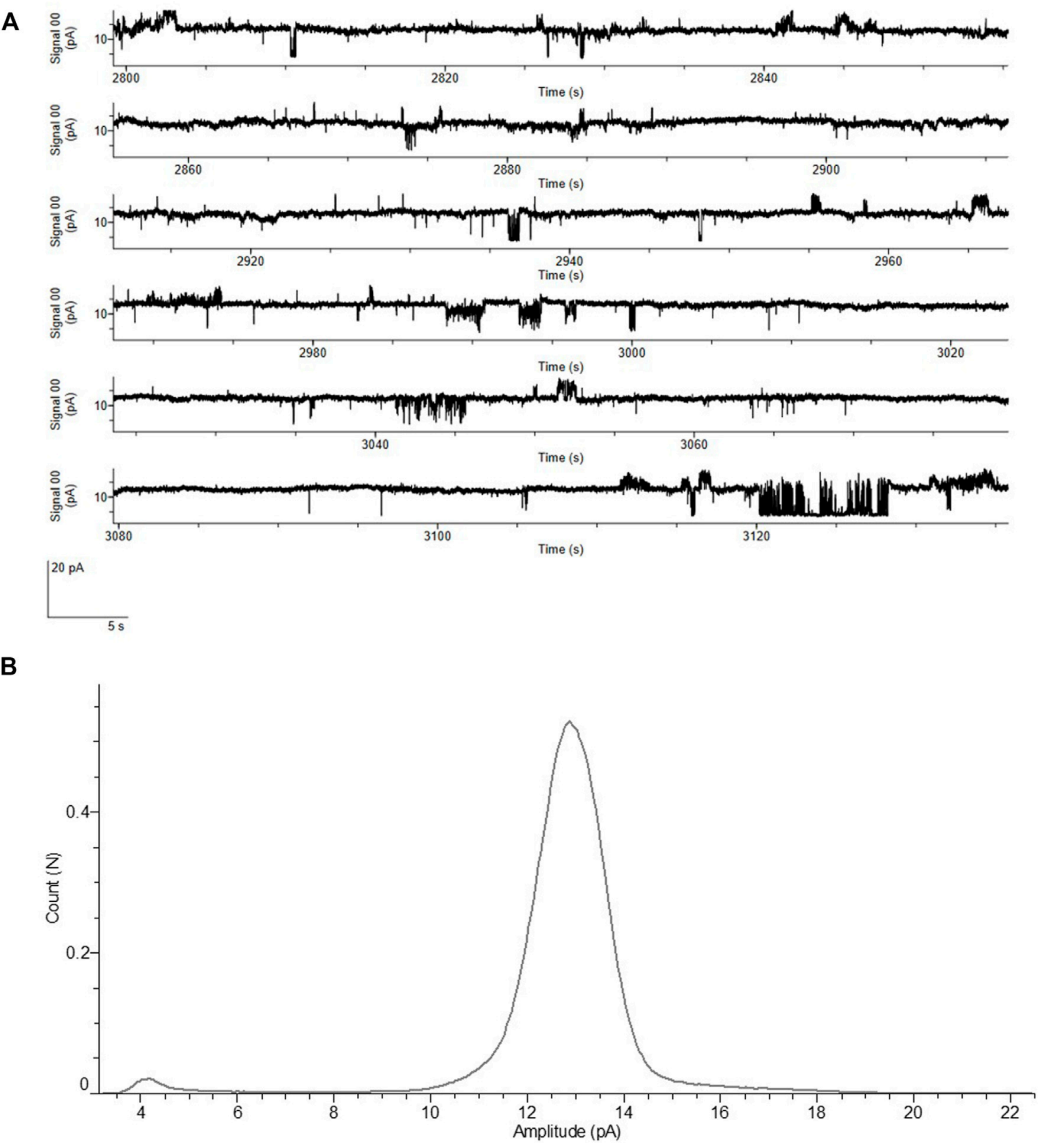
Functional analysis of ORF4 envelope protein reconstituted into lipid bilayer. **(A)** Current recordings at a voltage clamp of +100 mV from the ORF4 reconstituted into DPhPC bilayer. **(B)** All point Histogram plotted from the current recordings showing the large peak at 13 pA indicating a stable pore. All measurements of ORF4 were done in the presence of 150 mM NaCl, 10 mM HEPES, pH 7.0 buffer (*n* = 5).

Vaccine development is a key process once a novel pathogen presents itself, but the characterization of proteins harming the host as well as identifying potential blockers to the virus is of utmost importance. The ORF4 encoding the envelope protein might be a candidate for such blocking events as it is a viroporin (Xia et al., 2021). Therefore, we performed an in depth analysis of the envelope protein including the optimized cell-free protein synthesis (data not shown) as well as the characterization of the envelope protein as a cytotoxic channel like protein using planar lipid bilayer measurements. Different voltages were applied in order to measure the stable recordings from the protein reconstituted lipid bilayer. All the measurements were performed with the MF of the envelope protein. In order to avoid activity from the endogenous proteins in the microsomal vesicles, we used 150 mM NaCl as an electrolyte. Control measurements were performed with the same experimental conditions using the MF from the NTC without any expressed protein. Electrophysiological data of the envelope protein was compared to the controls. We noticed that there are small bilayer disruption currents leading to the lipid bilayer breaking in both cases, which we carefully ignored. There are two types of currents observed in the ORF4 reconstituted bilayers and completely absent in the controls. There is a single channel activity followed by pore formation in the ORF4 reconstituted lipid bilayer (Figure 7A and Supplementary Figure S2).

ORF4 reconstituted lipid bilayers showed different types of response ranging from cytotoxic events with large undefined current levels (>50 pA), pore forming stable currents of around 12–13 pA and single channel events with frequent flickering between open and closed states (5–7 pA). NTCs showed only few cytotoxic events and complete absence of pore and single channel events. The frequency of events is presented in Supplementary Table S1. Single channel events are further shown in Supplementary Figure S3. The histogram identified a large current peak at 13 pA suggesting a stable pore formation (Figure 7B). These data highlight the straightforward approach to analyze functionally active channel-like viral proteins derived from cell-free systems.

Overall, our data demonstrate that the CHO cell-free system can be applied to characterize the complete set of SARS-CoV-2 viral proteins and facilitates the screening of antibodies as well as pharmaceuticals and blockers against these viral proteins.

## Discussion

The rise of SARS-CoV-2 in late 2019 and the development to a global health issue in early 2020 continuing until today showed the dramatic results a viral outbreak could have. This has led to an increasing awareness worldwide to novel zoonotic diseases. The development of new detection techniques and a fast generation of adapted vaccines against virus variants are absolutely necessary as close interactions with wildlife and domesticated pets as well as climate change might pave the way for future pandemics (Scudellari, 2020; Beyer et al., 2021; Skegg et al., 2021; Thoradeniya and Jayasinghe, 2021). Until today some studies have evaluated the possibility of synthesizing viral proteins and virus-like proteins in cell-free systems. A virus like particle derived from the norovirus (Sheng et al., 2017) and Qβ phage (Smith et al., 2012) were synthesized in an *E. coli* based cell-free system. Using eukaryotic cell-free systems the capsid protein (Spearman and Ratner, 1996) and the envelope protein (Mikami et al., 2006) of the human immunodeficiency virus, the gp67 envelope protein (Merk et al., 2015), a virus like particle from the human papillomavirus (Wang et al., 2008) and the capsid protein of hepatitis E virus (Kubickova et al., 2021) have been synthesized and evaluated. These data show that cell-free protein synthesis can help to characterize single viral proteins and this knowledge can be used to further elucidate the complete virus. As cell-free protein synthesis uses a cell lysate rather than viable cells, no higher safety standards are needed for the synthesis of these viral proteins (Zemella et al., 2015). Our study aimed to demonstrate that cell-free protein synthesis can be applied to gain fast knowledge about individual novel viral proteins during a pandemic or epidemic, thus facilitating preparedness and responses.

Altincekic et al. (2021) demonstrated results on cell-free protein synthesis of ORF3, ORF6, ORF7a, ORF8, ORF9, the E and M protein in a wheat germ system and thus presented the first results on cell-free synthesis of SARS-CoV-2 proteins. These proteins were further purified and used for NMR spectroscopy. These data are extended by the findings acquired in this study as we showed that all different viral protein classes and groups could be synthesized the CHO lysate (Figures 1, 2). When looking at the nsp1, also called the leader protein, the binding of the 40S ribosome within the entry channel of the mRNA was shown which results in the inactivation of protein translation within the host (Thoms et al., 2020). Further data suggested the disruption of tRNA recruitment to the 80S ribosome (Banerjee et al., 2020). Banerjee et al. further showed that viral mRNA translation is not inhibited. As the *in vitro* protein translation was possible in the CHO-based system, this could also indicate that viral translation is not inhibited by nsp1. The activity of the protein was tested *in vitro* in HeLa and rabbit reticulocyte cell lysates as well as *in vivo* in HEK293T cells showing a downregulation in host protein translation of different proteins (Banerjee et al., 2020; Thoms et al., 2020). Our results depicted an inhibition of protein translation in an *in vitro* system based on CHO lysate indicating that different eukaryotic systems can be applied for the analysis of such a protein. Nonetheless, the effect on the protein synthesis of the model protein eYFP decreased over time and the fluorescence intensity was regained (Figure 3). In our system, the internal ribosomal entry site (IRES) from the cricket paralysis virus (CrPV) was used to initiate the translation independently from cap structures. Strikingly, a previous study showed that nsp1 associated less efficiently with the ribosomes when the CrPV IRES was used. The IRES from hepatitis C virus favored a binding of nsp1 (Lapointe et al., 2021). Thus, our data align with these prior findings by indicating that the effect of nsp1 was reduced by the CrPV IRES. Accordingly, future projects studying novel viral proteins should evaluate the protein synthesis and characterization in a cap-dependent and independent manner, as a protein translation could be detected in the CHO-based cell-free system.

After the successful synthesis and characterization of the nsp1 as a representative of the non-structural proteins, our study investigated the accessory proteins. Whilst the structure or function for some of the proteins is already known, other proteins yet have to be analyzed in detail. The ORF7b protein was already synthesized in a wheat germ cell-free system and multimerization could be shown (Fogeron et al., 2021). Here we demonstrate that ORF7a forms potential multimers up to a pentamer (Figure 4B). As this has not been shown before, detailed analysis of the multimeric state of the ORF7a has to be performed. ORF8 was shown to be involved in the immune invasion process (Flower et al., 2021) and structural analysis suggested a multimerization of the ORF8 via disulfide bridges (Flower et al., 2021). Additionally, potential glycosylation sites that stabilize the protein structure as was already seen in SARS ORF8ab were described (Mohammad et al., 2020). Accordingly, we analyzed ORF8 in our cell-free system in depth. Our data clearly depicted the formation of multimers even up to a tetramer of about 55 kDa suggesting stable disulfide bridges. In our CHO system a possible glycosylation of the monomer could be detected (Figure 4C). Future studies should evaluate whether the SARS-CoV-2 ORF8 is unstable without glycosylation as this was seen for SARS-CoV ORF8ab (Mohammad et al., 2020).

Until today, data on ORF10 are conflicting. Initial work on ORF10 identified epitopes for cytotoxic T lymphocytes and a possible alteration of SARS-CoV-2 pathogenicity by mutations in the ORF10 protein was described (Hassan et al., 2021). Other studies reflected that the protein can be terminated prematurely but also read through was emphasized (Pancer et al., 2020; Schuster, 2020). Within our work we could show the synthesis of ORF10 in the presence of a signal peptide (Figure 4). We also present oligomerization of ORF10 (Figure 1B) which correlates to previous work where it was hypothesized that ORF10 might oligomerize to form a pore for ion fluctuations co-localizing with other accessory proteins (Schuster, 2020). The data seen in this study clearly show that the translation initiation was improved in the presence of a signal peptide. This could indicate a better sequence context for protein translation. In prior studies, it was further suggested that ORF10 binds to an E3 ubiquitin ligase and the N-terminus is essential for that matter, but it was still not important for the infection by SARS-CoV-2 (Mena et al., 2021). Thus, our data depict a stable synthesis of ORF10 only in the presence of a N-terminal signal peptide supporting the importance of the N-terminus in ORF10 for structure and function.

Structural proteins of a viral pathogen are the first proteins to be analyzed in detail as these proteins are mainly used for primary response mechanisms. Therefore, it was mandatory to show that our cell-free system can be used to study and evaluate these proteins. In particular, we chose the nucleocapsid protein to validate our cell-free system for the detection of viral proteins in an in-solution ELISA, a Western Blot and a Dot Blot (Figure 6). Prior work has shown that Dot Blots are regularly used for the detection of SARS-CoV-2 antibodies in patient samples. In a Dot Blot, the complete nucleocapsid protein as well as nucleocapsid protein fragments were able to identify SARS-CoV-2 positive patient samples (Smits et al., 2021). In our study, we showed that cell-free synthesized proteins can be spotted and detected at different protein concentrations and therefore we can qualify cell-free protein synthesis for the validation of antibodies. We showed that the N protein as well as early onset mutations could be detected. The results were obtained using an anti-SARS-CoV-2 and anti-SARS antibody reflecting on the possibility of cell-free systems to assist in tackling future pandemics right from the beginning.

Finally, our study aimed to further characterize the envelope protein. As the envelope protein from the SARS outbreak in 2003 is a homo-pentameric cation channel (Wilson et al., 2004; Verdiá-Báguena et al., 2012), it was suggested that the SARS-CoV-2 envelope protein reflects a similar structure and mechanism of action. In our study, we could also show the pentameric state of the SARS-CoV-2 envelope protein after the cell-free synthesis (Figure 5A). A channel-like structure forming a pore of 2.1 Å has already been depicted (Mandala et al., 2020) and cytotoxic activity on various cell lines was shown (Xia et al., 2021) in initial studies. In a recent study the activity of the envelope protein as a cation channel potentially selective for potassium, sodium, calcium and magnesium was demonstrated in planar lipid bilayer recordings (Xia et al., 2021). The cation channel like behavior and pH sensitivity was further observed in *Xenopus* oocytes (Cabrera-Garcia et al., 2021). These data reflect upon the envelope protein as a drug target. In addition to the just mentioned studies, the envelope protein showed different types of electrophysiological activities in the presence of a lipid bilayer. Single channel activity typical for ion channels with transitions between open and closed states and with a stable baseline current could be detected. Sometimes these single channel events were followed by a pore formation with stable currents of around 13 pA at +100 mV without any flickering activity. This could be due to the complete pentameric assembly of the protein leading to an active open pore. Currents, which are large and abrupt and do not resemble the typical ion channel activity, were frequently observed. They are typically measured for cytotoxic proteins. These large cytotoxic currents (>50 pA) might be due to the increase in the surface density of the envelope protein assembly leading to membrane destabilization. Therefore, our data combine the knowledge gathered in previous studies. It was already shown that the envelope protein from other viral infections such as MERS and SARS demonstrated single-channel activity in lipid bilayers suggesting a pentameric ion channel (Surya et al., 2015). Whole cell recordings of SARS envelope protein expressed in HEK293 cells indentified a cation selective channel like behavior (Pervushin et al., 2009). Data on the SARS-CoV-2 envelope protein in combination with data on MERS and SARS envelope proteins reflect upon the fact that the ORF4 envelope protein from SARS-CoV-2 is a cation selective ion channel that triggers cytotoxic events. Thus, blocking the envelope protein of SARS-CoV-2 could inhibit pathogenic events in the host. To our knowledge, this is the first study where a viroporin could directly be analyzed in planar lipid bilayer recordings when synthesized in a cell-free system without any additional purifications.

As discussed, novel pathogens could develop easily provoking challenges in modelling the emergence of novel pathogens. Moreover, the length of a pandemic cannot be predicted in general as diverse factors play a role in the progression of the infection (Scudellari, 2020). It is necessary to identify the most promising prevention methods, but therapeutic approaches are further needed (Meganck and Baric, 2021). The results obtained in this study qualify cell-free systems as a rapid response methodology for evidence-informed decision making in health policy and research. All viral proteins of SARS-CoV-2 could be synthesized, studied and proteins like the envelope protein could be functionally characterized. Antibody interaction with cell-free produced viral proteins was demonstrated and we have further shown that mutants of the nucleocapsid protein, can be assessed. The omicron variant of SARS-Cov-2 has shown that mutations can alter the course of the pandemic (Karim and Karim, 2021). Therefore, a rapid system for the characterization of viral variants is necessary. As presented in this study, 2-step PCR reactions were used to modify the templates for the cell-free reactions. Thereby demonstrating that a simple cloning-free method to efficiently generate templates suitable for coupled cell-free protein production was applied for viral genes. Based on previous studies and on the data presented here (Sawasaki et al., 2002; Bechlars et al., 2013), templates for viral mutants can be designed by fast and efficient 2-step PCR schemes thus allowing for the rapid and parallel high-throughput screening of the functional activity of mutants. This procedure will further allow the screening of antibodies and potential inhibitors against mutant viral proteins without using BSL-1 through 4 laboratories.

Taken together, we identified eukaryotic cell-free systems as a versatile technology to synthesize and characterize the viral pathogen SARS-CoV-2. The data presented here demonstrate that eukaryotic cell-free systems facilitate the rapid response to novel zoonotic diseases.

## Materials and Methods

### Template Design

Plasmids encoding each ORF or single protein of SARS-CoV-2 genome (MN908947.3) were designed according to Brödel et al. (2013) containing the internal ribosomal entry site (IRES) of the cricket paralysis virus (CrPV) and the T7-promoter (Brödel et al., 2013). Nucleocapsid mutations were designed accordingly based on MT081067.1 (ASN202, exchange at amino acid 202 from serine to asparagine) and MT123290.1 (SER343, exchange at amino acid 343 from proline to serine) Genes were obtained by *de novo* gene synthesis (Biocat GmbH) in a pUC57-1.8K vector backbone. These plasmids were directly used for cell-free protein synthesis.

The full length Spike protein was also obtained by *de novo* gene synthesis (Biocat GmbH) in a pCI vector. This gene was amplified using an expression PCR (E-PCR) with the HiFidelity polymerase (Qiagen). Standard PCR protocol was applied using the forward N0 (5′ ATG ATA TCT CGA GCG GCC GCT AGC TAA TAC GAC TCA CTA TAG GGA GAC CAC AAC GGT TTC CCT CTA GAA ATA ATT TTG TTT AAC TTT AAG AAG GAG ATA AAC AAT G 3′) and reverse C0 (5′ ATG ATA TCA CCG GTG AAT TCG GAT CCA AAA AAC CCC TCA AGA CCC GTT TAG AGG CCC CAA GGG GTA CAG ATC TTG GTT AGT TAG TTA TTA 3′) primers.

As ORF3 is a putative channel-like protein the influence of the Mel signal peptide on the possible membrane embedding and multimerization was investigated. As only an NC construct was available for the ORF3, a 2-step EPCR was performed to fuse the Mel signal peptide to the ORF3 gene based on Bechlars et al. (2013). Shortly, in a first step the gene specific forward primer X-CoV-2-ORF3-oe-Mel-F (5′ TAC ATT TCT TAC ATC TAT GCG GAC GAT TTG TTT ATG AGA ATC TT 3′) and the reverse adapter primer C0 were used. During this step an overlap from the ORF3 DNA template to the Mel signal peptide was generated. In the second step, the adapter primers NCM-F (5′ ATG ATA TCT CGA GCG GCC GCT AGC TAA TAC GAC TCA CTA TAG GGA GAC CAC AAC GGT TTC CCT CTA GAA ATA ATT TTG TTT AAC TTT AAG AAG GAG ATA AAC AAA AGC AAA AAT GTG ATC TTG CTT GTA AAT ACA ATT TTG AGA GGT TAA TAA ATT ACA AGT AGT GCT ATT TTT GTA TTT AGG TTA GCT ATT TAG CTT TAC GTT CCA GGA TGC CTA GTG GCA GCC CCA CAA TAT CCA GGA AGC CCT CTC TGC GGT TTT TCA GAT TAG GTA GTC GAA AAA CCT AAG AAA TTT ACC TGC TAA ATT CTT AGT CAA CGT TGC CCT TGT TTT TAT GGT CGT ATA CAT TTC TTA CAT CTA TGC GGA C 3′) and the C0 reverse primer were used according to standard protocol. As a final construct, the NCM-ORF3-C0 was generated where a Mel signal peptide was fused to the ORF3 gene.

### Cell-Free Protein Synthesis

Cell-free synthesis reactions using translationally active lysate derived from eukaryotic cells were performed as previously described by utilizing Chinese hamster ovary cells (CHO-K1) (Brödel et al., 2014; Thoring et al., 2016). Further, each template was applied individually and a no template control (NTC) consisting of a translation mixture without any DNA template was used as a background control.

### Batch-Based Reactions

Protein synthesis was conducted in coupled transcription/translation reactions in a final volume of 25–80 μl. Cell-free synthesis reactions were composed of 40% (v/v) translationally active CHO lysate supplemented with HEPES-KOH (pH 7.6, f.c. 30 mM, Carl Roth GmbH), sodium acetate (f.c. 100 mM, Merck), Mg(OAc)_2_ (f.c. 3.9 mM, Merck), KOAc (f.c. 150 mM, Merck), amino acids (complete 100 μM, Merck), spermidin (f.c. 0.25 mM; Roche), Dithiothreitol (DTT, 2.5 mM, Life technologies GmbH) and energy regenerating components including creatine phosphokinase (f.c. 0.1 mg/ml, Roche), creatine phosphate (20 mM, Roche), ATP (1.75 mM, Roche) and GTP (0.3 mM, Roche). To allow for DNA transcription during cell-free protein synthesis, 1 U/μl T7 RNA polymerase, 0.3 mM of UTP (Roche) and CTP (Roche) and 0.1 mM of the cap analogue m7G(ppp)G (Prof. Edward Darzynkiewicz, Warsaw University, Poland) were added to the reaction. Additionally, PolyG primer (f.c. 12 µM, IBA) was supplemented. To monitor the protein quantity and quality, cell-free protein synthesis reactions were supplemented with radioactive ^14^C-leucine (f.c. 50 μM, specific radioactivity 66.67 dpm/pmol, Perkin Elmer). Batch synthesis reactions were incubated at 30°C for 3 h at 500 rpm (Thermomixer comfort, Eppendorf).

### Continuous-Exchange Cell-Free Reactions

CECF-reactions were performed as previously described (Thoring et al., 2017). Briefly, reactions took place in commercially available dialysis devices (Biotechrabbit GmbH) and incubated in a thermomixer (Eppendorf) for 24–48 h, at 30°C at 600 rpm. Two mixtures were individually prepared. Reaction mixtures were composed similar to batch-based reactions (see above). PolyG was added at a final concentration of 4.5 µM and Mg(OAc)2 was added at a final concentration of 18.5 mM. The feeding mixture was composed of HEPES-KOH (f.c. 30 mM, pH 7.6), Mg(OAc)2 (f.c. 3.9 mM), KOAc (f.c. 150 mM), amino acids (complete 100 µM f.c.), spermidine (f.c. 0.25 mM), energy regenerating components (f.c. 1.75 mM ATP, 0.3 mM GTP), CTP (f.c. 0.3 mM), UTP (f.c. 0.3 mM) and the cap analogue G(ppp)G (f.c. 0.33 mM). Further, the caspase inhibitor AC-DEVD-CMK (f.c. 30 µM; Promega) and sodium azide (f.c. 0.02%, Merck) were added to both reaction and feeding mixture. ^14^C-leucine (f.c. 50 μM, specific radioactivity 10 dpm/pmol) for radio-labeling of *de novo* produced proteins was added to the reaction, when necessary.

### Fractionation

After the incubation time the crude translation mixture (TM) was centrifuged (16,000xg, 10 min, 4°C) resulting in the supernatant (SN), containing the soluble subunits, and the pelleted microsomes containing putative membrane bound subunits. The pellet was resuspended in phosphate buffered saline (PBS) resulting in the microsomal fraction (MF).

### Analysis of Radio-Labeled Proteins

Total protein yields of cell-free synthesized proteins were determined by incorporation of ^14^C-leucine and subsequent precipitation by hot trichloro acetic acid (TCA, Carl Roth GmbH). Briefly, 3–5 µl aliquots of the fraction of interest were mixed with 3 ml of 10% TCA/2% casein hydrolysate (Carl Roth GmbH) solution and incubated at 80°C for 15 min. After a 30 min incubation on ice, ^14^C-labeled proteins were transferred to membrane filters (VWR) using a vacuum filtration system (Hoefer). Filters were washed with 5% TCA to remove non-incorporated ^14^C-leucine and dried with acetone. Subsequently, filters were placed in 3 ml scintillation cocktail (Quicksafe A, Zinsser Analytik), incubated for at least 1 h and measured by liquid scintillation counting using the Hidex 600 SL (Hidex). Total protein yields of *de novo* synthesized proteins were calculated based on the molecular weight and number of leucines of the respected protein.

For qualitative analysis of the proteins’ homogeneity and molecular size, 3–5 µl aliquots were precipitated in cold acetone (Carl Roth GmbH) as described previously (Thoring et al., 2017). Sodium dodecyl sulfate polyacrylamide gel electrophoresis (SDS-PAGE) using precast gels (NuPAGE, 10% Bis-Tris, Life technologies) and self-prepared 10% gels using SureCast resolving and stacking buffer (Thermo Fisher Scientific) was performed. The SeeBlue Pre-Stained marker (Thermo Fisher) was used as a standard for the molecular weight measurement. Precast gels were run at 185 V for 35 min while self-prepared gels were run at 150 V for 55 min. Gels were dried at 70°C (Unigeldryer 3545D, Uniequip, Planegg), placed on a phosphor screen (GE Healthcare) and radioactively labeled proteins were visualized using a Typhoon Trio + variable mode imager (GE Healthcare).

### Protein Translation Inhibition Caused by nsp1

Nsp1 was pre-synthesized in a cell-free manner in a CHO lysate and subsequently added to the synthesis of a non-viral model protein, namely an enhanced yellow fluorescent protein (eYFP). Defined concentrations of the nsp1 were administered to the cell-free synthesis of eYFP. The NTC was administered as a volume equivalent to the highest nsp1 concentration. A 50 µl reaction of the eYFP was pipetted in a black 96-well plate and placed into a microplate reader (Mithras Tristar^2^ LB 943 Berthold Technologies). The fluorescence intensity of the eYFP was measured every 10 min during the 3 h synthesis time.

### Deglycosylation Assay

Protein N-glycosylation was investigated using PNGaseF (peptide N-glycosidase F, NEB) or EndoH (endoglycosidase H, NEB) according to the manufacturer’s protocol. Therefore, proteins were synthesized in a cell-free manner in the presence of ^14^C-leucine. Subsequently, 5 µl of the protein sample were used for the deglycosylation assay and precipitated in acetone for SDS-PAGE analysis as described above.

### In-Solution ELISA

Cell-free synthesized nucleocapsid proteins were mixed with Ni-NTA Magnetic Agarose Beads (Qiagen) and handled according to the manufacturer’s protocol. Shortly, samples were incubated with beads over night at 4°C on a rotator. All following steps were performed on ice. The next day, beads were washed three times and 200 µl of primary anti-nucleocapsid antibody (1:1000 in binding buffer, Antibodies online ABIN6953059) were added to the beads and incubated at 4°C on a rotator for 2 h. This washing step and antibody incubation was repeated for the secondary HRP-linked anti-rabbit antibody (1:3000 in binding buffer, Cell Signaling Technologies). After a final washing procedure, 100 µl TMB-solution was added to the beads and incubated on ice for 1 min. The TMB solution was separated from the beads and mixed with 100 µl H_2_SO_4_. The absorbance at 450 and 620 nm was measured using the Mithras Tristar^2^ LB 943 (Berthold Technologies).

### Western Blot

For qualitative binding analysis, the SN fractions of the nucleocapsid WT proteins and the two mutants were analyzed. Proteins were synthesized in a cell-free manner using the CHO eukaryotic lysate. Samples were precipitated with ice cold acetone and run on a self-cast SDS-PAGE as described above. The SeeBlue Plus 2 Pre-Stained marker (Thermo Fisher) was used as a weight measurement. Subsequently, Western blotting was performed using the iBlot Gel transfer device (Invitrogen) where proteins were blotted onto a PVDF membrane with 20 V for 10 min. Membranes were washed with Tris buffered saline with 0.1% Tween (TBS/T) for 5 min, repeated twice and incubated overnight at 4°C with blocking buffer (2% BSA in TBS/T). On the next day, the membranes were washed three times in TBS/T before incubating the membrane with the commercially available antibody against the SARS nucleocapsid protein sc58193 and SARS-Cov-2 N protein ABIN6953059 (antikoerper-online.de and Santa Cruz Biotechnology, respectively, 1:1,000, 1% BSA in TBS/T) for 3 h at room temperature on an orbital shaker at 60 rpm. The washing procedure in TBS/T was repeated again, followed by an incubation of 2 h with the secondary HRP-linked anti-mouse IgG antibody (1:3000, 1% BSA in TBS/T, Cell Signaling Technologies). The binding was visualized with chemiluminescent WesternBright ECL HRP substrate (Advanstra) and detected with the Azure c600 Gel Imaging System (Azure Biosystems).

### Dot Blot Analysis

The total protein yield of cell-free synthesized nucleocapsid protein was determined by hot TCA precipitation as described above. Defined yields of 15, 10, 5, 1 ng protein were precipitated in ice cold acetone (see above) and the dried pellet was solubilized in 4 µl LDS buffer and 1 µl protein binding buffer (G-Biosciences, VWR). Subsequently, the Enhancer Dot Blot System (G-Biosciences, VWR) was used to spot the protein samples onto a nitrocellulose membrane (GE Healthcare). After that the same washing, blocking and antibody incubation procedure as for the Western Blot analysis was performed. Primary antibody against the SARS-Cov-2 N protein ABIN6953059 (antikoerper-online.de) in a 1:1000 dilution. The HRP-linked anti-mouse secondary antibody was used in a 1:3000 dilution. Again, the binding was visualized with chemiluminescent WesternBright ECL HRP substrate (Advanstra) and detected with the Azure c600 Gel Imaging System (Azure Biosystems).

### Electrophysiological Analysis of the ORF4 Envelope Protein

Planar bilayer experiments were performed as explained previously (Thoring et al., 2017). Lipid bilayers were formed from 1,2-diphytanoyl-sn-glycero-3-phosphocholine (DPhPC, (Avanti Polar Lipids) which were dissolved in octane (Sigma Aldrich) at a concentration of 10 mg/ml. Concentrations of 150 mM sodium chloride (NaCl, Sigma Aldrich), 10 mM HEPES (Sigma Aldrich), buffered at pH 7.0 were used as an electrolyte. For current measurements, voltage ramp protocol, as well as individual voltage steps were applied, to analyze the functional properties. A single channel amplifier (EPC-10, HEKA Electronic Dr. Schulze GmbH) was connected to the multiplexer electronics port of the Orbit16 system (Nanion). Recordings were done at a sampling rate of 50 kHz with a 10 kHz Bessel. Data were analyzed with Clampfit (Molecular Devices). 5 µl of MF of the cell-free synthesized envelope protein were added into the chamber containing 180 µl of the buffer and mixed gently by pipetting for a better fusion of microsomes with the underlying lipid bilayer. After waiting for 20 min electrophysiological measurements were started.

## Data Availability

The datasets presented in this study can be found in online repositories. The names of the repository/repositories and accession number(s) can be found below: https://www.ncbi.nlm.nih.gov/nuccore/MN908947.3.
