# Supplementary material for: The Potential of Eukaryotic Cell-Free Systems as a Rapid Response to Novel Zoonotic Pathogens: Analysis of SARS-CoV-2 Viral Proteins
Source: Front Bioeng Biotechnol. 2022 Apr 19;10:896751. doi: 10.3389/fbioe.2022.896751 (PMC9061942; doi:10.3389/fbioe.2022.896751)
Supplement: Supplementary file 1 [file DataSheet1.docx]

Supplementary Material

# Supplementary Figures and Tables


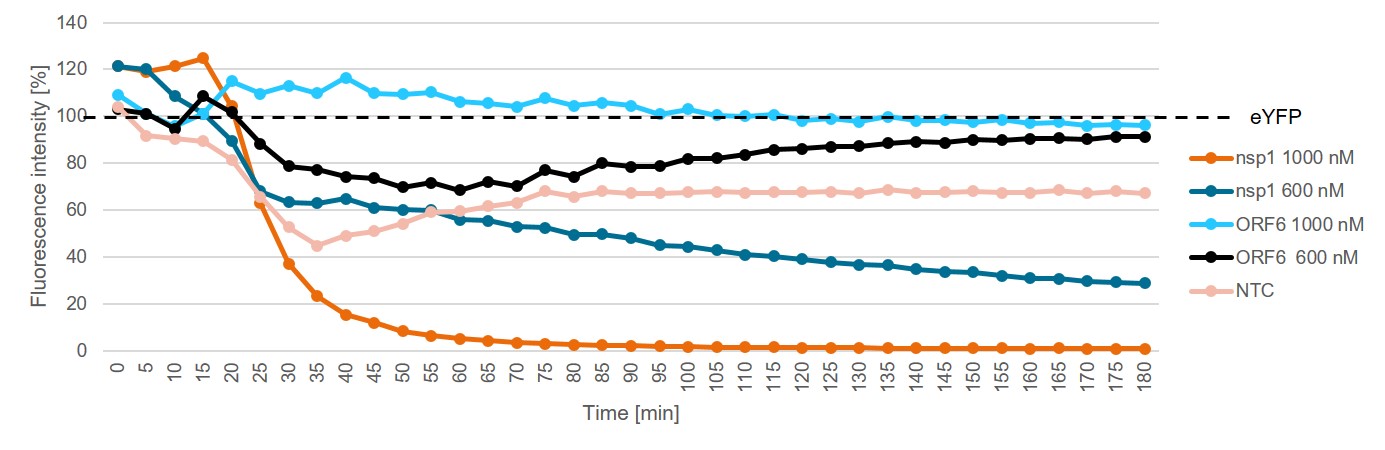


**Supplementary Figure 1.** Nsp1 inhibitory effect. Initial test of nsp1, ORF6 control protein and an NTC on the cell-free protein synthesis of the model protein eYFP detected by the fluorescence intensity (%). Data were normalized to eYFP fluorescence signal.

**Supplementary Table 1**. Events measured from both the ORF4 envelope reconstituted lipid bilayers and no template control (NTC)

|  | **ORF4 (n= 5)** | **NTC (n=3)** |
| --- | --- | --- |
| Pore / Cytotoxic events | 18 | 3 |
| Single channel events | 7 | 0 |


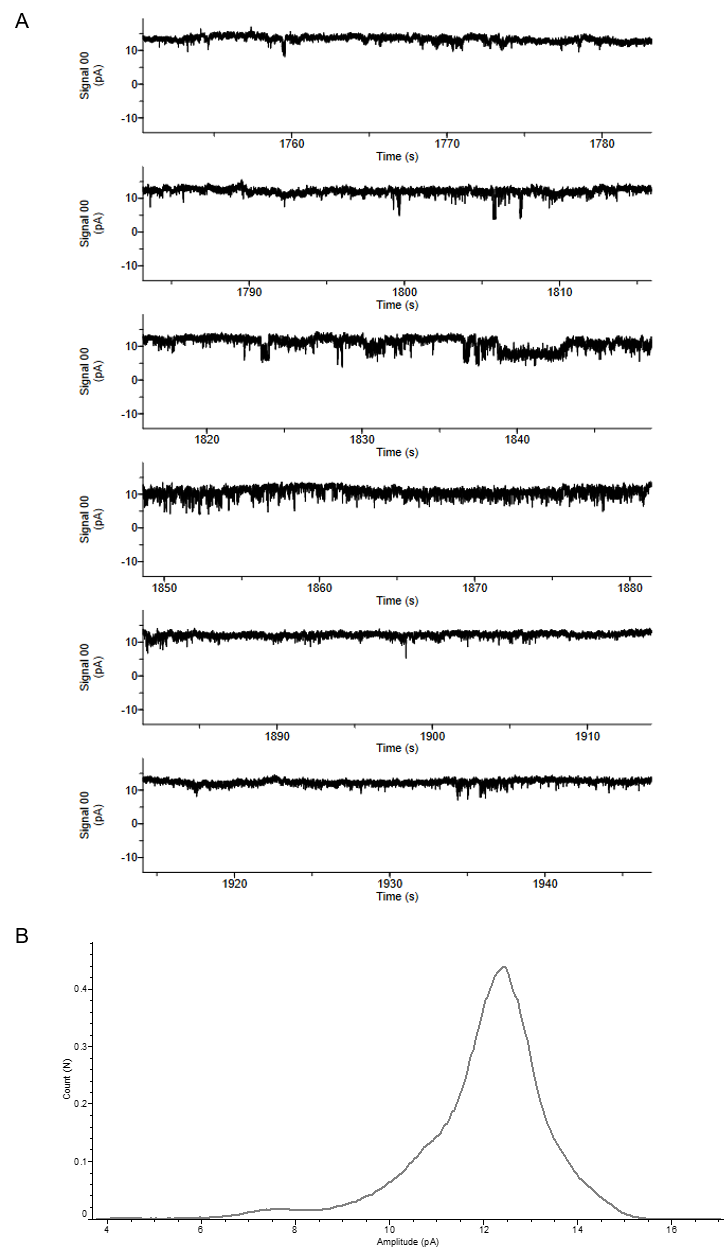


**Supplementary Figure 2.** Functional analysis of ORF4 envelope protein reconstituted into lipid bilayer. (A) Current recordings at a voltage clamp of + 100mV from the ORF4 reconstituted into DPhPC bilayer. (B) All point histogram plotted from the current recordings showing the large peak at 13 pA indicating a stable pore. All measurements were done in the presence of 150 mM NaCl, 10 mM HEPES,pH 7.0 buffer (n = 5).


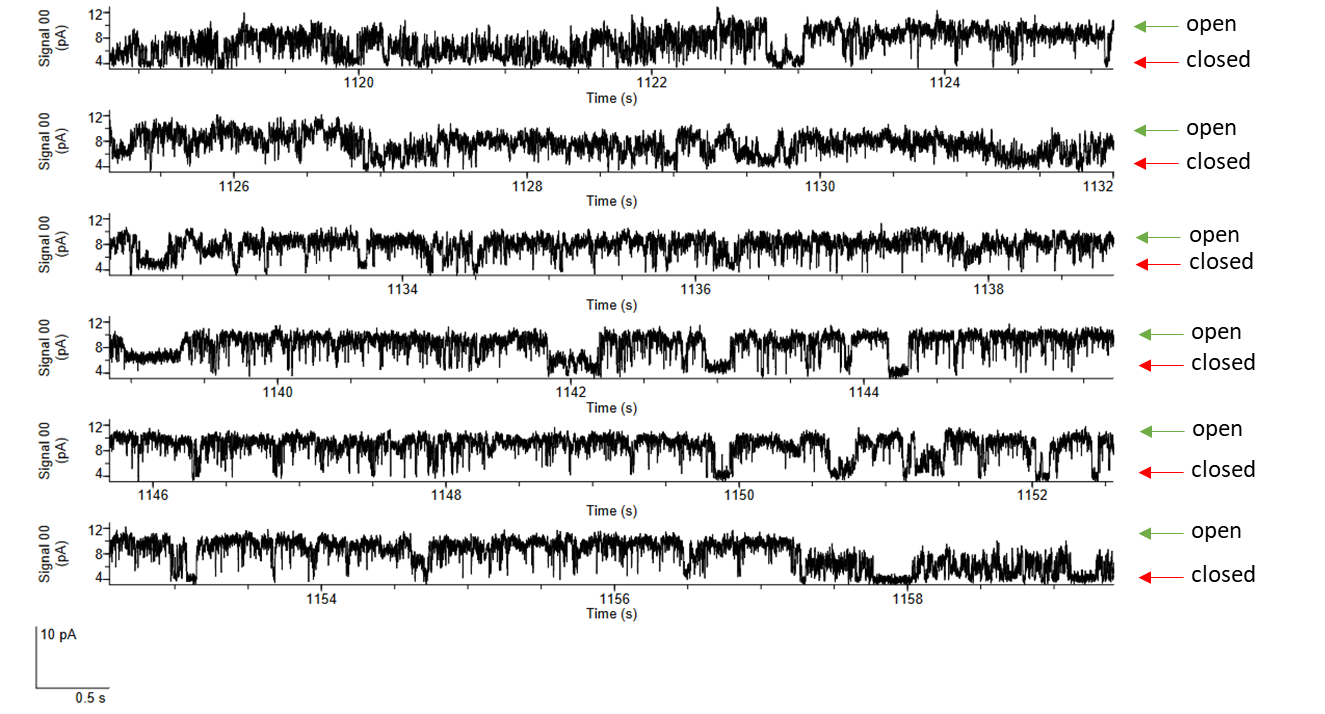


**Supplementary Figure 3.** Single channel observed from the ORF4 envelope protein reconstituted into lipid bilayer. Current recordings showing current transitions between open and closed states at a voltage clamp of + 100mV from the ORF4 reconstituted into DPhPC bilayer. All measurements were done in the presence of 150 mM NaCl, 10 mM HEPES,pH 7.0 buffer (n = 5).
